# Latitudinal Variation of a Defensive Symbiosis in the *Bugula neritina* (Bryozoa) Sibling Species Complex

**DOI:** 10.1371/journal.pone.0108783

**Published:** 2014-10-02

**Authors:** Jonathan Linneman, Darcy Paulus, Grace Lim-Fong, Nicole B. Lopanik

**Affiliations:** 1 Department of Biology, Georgia State University, Atlanta, Georgia, United States of America; 2 Department of Biology, Randolph-Macon College, Ashland, Virginia, United States of America; CSIR- National institute of oceanography, India

## Abstract

Mutualistic relationships are beneficial for both partners and are often studied within a single environment. However, when the range of the partners is large, geographical differences in selective pressure may shift the relationship outcome from positive to negative. The marine bryozoan *Bugula neritina* is a colonial invertebrate common in temperate waters worldwide. It is the source of bioactive polyketide metabolites, the bryostatins. Evidence suggests that an uncultured vertically transmitted symbiont, “*Candidatus* Endobugula sertula”, hosted by *B. neritina* produces the bryostatins, which protect the vulnerable larvae from predation. Studies of *B. neritina* along the North American Atlantic coast revealed a complex of two morphologically similar sibling species separated by an apparent biogeographic barrier: the Type S sibling species was found below Cape Hatteras, North Carolina, while Type N was found above. Interestingly, the Type N colonies lack “*Ca.* Endobugula sertula” and, subsequently, defensive bryostatins; their documented northern distribution was consistent with traditional biogeographical paradigms of latitudinal variation in predation pressure. Upon further sampling of *B. neritina* populations, we found that both host types occur in wider distribution, with Type N colonies living south of Cape Hatteras, and Type S to the north. Distribution of the symbiont, however, was not restricted to Type S hosts. Genetic and microscopic evidence demonstrates the presence of the symbiont in some Type N colonies, and larvae from these colonies are endowed with defensive bryostatins and contain “*Ca.* Endobugula sertula”. Molecular analysis of the symbiont from Type N colonies suggests an evolutionarily recent acquisition, which is remarkable for a symbiont thought to be transmitted only vertically. Furthermore, most Type S colonies found at higher latitudes lack the symbiont, suggesting that this host-symbiont relationship is more flexible than previously thought. Our data suggest that the symbiont, but not the host, is restricted by biogeographical boundaries.

## Introduction

The biogeographical clines and boundaries that define the geographical distribution of an organism have been studied for many metazoans [1]–[3]. The importance of these limits to the distribution of symbionts of those metazoans and the partners together (the holobiont), however, has rarely been thoroughly explored. While many symbiotic relationships are considered mutualisms, in which both partners benefit, there can be physiological costs associated with hosting a symbiont [4]. Beneficial defensive symbionts can facilitate the survival of the host against enemies such as pathogens, competitors, or predators [5]. Often, these enemies are ephemeral, or their import varies among populations. In the absence of the selective pressure, the costs of hosting a symbiont may eclipse the potential benefits of a partnership, resulting in an unpartnered host with greater fitness than a partnered host [6]–[8]. Selection over time would then ultimately result in symbiont loss. Defensive symbionts that are vertically transmitted are thought to represent a significant parental investment. This implies both that the symbionts are beneficial to the host, and that the host and symbiont have developed mechanisms to prevent loss of the symbiont; therefore, symbiont loss represents an irreversible evolutionary milestone. The increase in aposymbiotic host frequency in the absence of the enemy would then suggest that the symbiont imposes a cost on its host (reviewed in [9]). The interplay of symbiont cost-benefit and biogeography is likely important to host/symbiont partners with widespread distribution, but is not well understood.

The marine bryozoan genus *Bugula* provides an interesting platform for the exploration of the importance of geographic variation to symbiotic interactions. Among commonly studied *Bugula* species, four are known to harbor bacterial symbionts seemingly absent from at least two congeners [10]–[13]. These closely related symbionts do not appear to have a history of strict cospeciation with their hosts, although vertical transmission is suspected due to their presence in the larvae and larval brood chambers, or ovicells, of colonies [14]. This suspected vertical transmission has also led to a high degree of congruence between the phylogenetic topologies of host and symbiont [11]. Within the genus, the cryptic species complex of the cosmopolitan bryozoan *Bugula neritina* allows for even more direct exploration of the ties between symbiosis and host ecology. *Bugula neritina* is known to harbor a *γ*-Proteobacterial symbiont that is, to date, uncultivated [10], [15]. “*Candidatus* Endobugula sertula” is found in all life stages of the host: in larvae, it is found in a circular groove located along the aboral pole called the pallial sinus [10], [15]; in adult colonies, it is found in funicular cords, which serve as a vascular system, transporting nutrients and wastes throughout the colony [16] and to a developing embryo in an ovicell [14].

Evidence from antibiotic-curing experiments suggests that “*Ca.* Endobugula sertula” is the source of the bryostatins [17], [18], complex bioactive polyketides with medical potential (reviewed in [19]). Feeding assays with extracts of *B. neritina* and purified bryostatins revealed that these symbiotically produced compounds are unpalatable and may serve to protect the vulnerable host larvae from predators, suggesting an association in which bryostatin presence may drive maintenance of symbiosis over generations. Both *B. neritina* larvae and larval extracts are unpalatable to co-occurring invertebrate and vertebrate predators including oysters [20], corals, sea anemones, filefish, and the pinfish, *Lagodon rhomboides* [18], [21]–[23]. Larvae have also shown a marked ability to survive and metamorphose after rejection by predators, with>90% success after rejection by the coral *Oculina arbuscula* [21] and 100% metamorphosing after attacks by *L. rhomboides* and the filefish *Monocanthus ciliatus* [23]. Importantly, the defensive bryostatins appear to be most concentrated in the larval stage of the *B. neritina* life cycle [18], [22], [23], suggesting that alternative protective means such as structural defenses may be more crucial to adult colonies. The *B. neritina*-“*Ca.* Endobugula sertula” association is one of the most well-characterized examples of a marine system involving defensive natural products that are produced by a microbial symbiont [5]. At least one terrestrial analog has been identified, in which the polyketide pederin, produced by a bacterial endosymbiont, prevents predation on the larval stage of the rove beetle genus *Paederus* [24], [25].

In the case of *B. neritina*, while the presence of defensive metabolites suggested that the association with “*Ca.* Endobugula sertula” may provide a distinct advantage for larval survival, some populations of *B. neritina* were shown to lack these endosymbionts and, consequently, the suite of bryostatins associated with them [18], [26]. Further genetic characterization over the past several years has identified three cryptic sibling species of *B. neritina* based upon the sequence of the mitochondrial cytochrome *c* oxidase I (COI) gene: (1) Type S (“Shallow”) found in temperate environments worldwide, (2) a Type D (“Deep”) variety found only on the Eastern Pacific coast, and so named due to its occurring at depths 9 m or greater in southern California, and (3) Type N (“Northern”) found only on the Western Atlantic coast, initially in Delaware and Connecticut [12], [26], [27]. These so-called “sibling species” have recently been proposed as distinct biological species [28].

Types S and D *B. neritina* house closely related bacterial symbionts which differ at just four nucleotide sites (0.4% of the 996-bp shared sequenced region) in the small subunit ribosomal RNA gene (16S rRNA). Interestingly, bryostatin composition has been shown to vary between the Type S and D populations, which could be attributed to either host or symbiont [12]. The *bry* gene cluster, which putatively prescribes bryostatin biosynthesis, shows minimal variation between the sibling species, demonstrating 98% identity and differing most significantly in the genomic location of several accessory genes [29]. Perhaps most notably, Type N populations initially gave no evidence of endosymbionts or of bryostatin production. Although extracts of Type N *B. neritina* were shown to be significantly unpalatable to a predator from North Carolina, decreasing palatability by nearly 40%, these Type N samples were not as deterrent as extracts with bryostatins, which reduced feeding by 80% [18]. Because of this pattern of palatability and of sibling species location, researchers proposed a biogeographic division on the North American east coast between the ranges of Type N and S *B. neritina*, with the traditional boundary Cape Hatteras [30]–[32] initially speculated as a point of transition. In this separation of sibling species along the Western Atlantic coast, survival of Type S *B. neritina* in lower-latitude waters would be enabled by maintenance of the symbiosis and associated defensive metabolites in the presence of higher predation pressure [33]. Its spread northward, meanwhile, is limited by environmental tolerance limits of the Type S bryozoan or its symbiont [22], [26].

In 2010, we began assessing the genetic and chemical composition of *B. neritina* populations along the United States' east coast with the goal of uncovering differences in local bryostatin composition and symbiont identity. We discovered that *B. neritina* sibling species distribution on both sides of Cape Hatteras was inconsistent with previous reports. Type N *B. neritina* were found in two populations in South Carolina, previously thought to be exclusively Type S, while Type S colonies were located as far north as Maryland. Furthermore, the previously assumed aposymbiotic Type N *B. neritina* was associated with a bacterial symbiont at low latitudes, while Type S colonies north of Cape Hatteras surprisingly lacked this association. These initial findings led us to examine *B. neritina* populations along the Atlantic coast more closely in order to better understand the geographic and phylogenetic relationship of Type N and Type S, as well as to correlate varying symbiotic and bryostatin status with the distribution of both sibling species.

## Materials and Methods

### Sampling of coastal *B. neritina* populations and extraction of DNA

**Figure 1:**
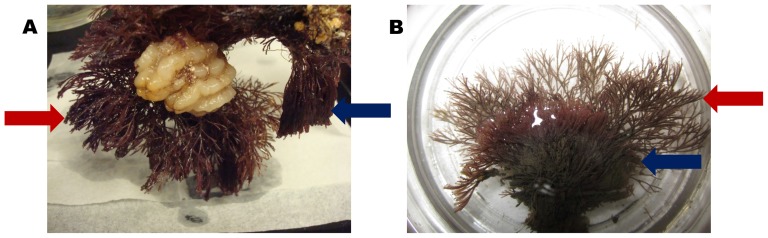
Co-occurrence of Type N and Type S *B. neritina*. Colonies collected together in (A) Beaufort, NC, and (B) Oyster, VA. Blue arrows indicate Type N colonies; red indicate Type S.

Adult *B. neritina* colonies were collected from floating docks in coastal locations ranging from Indian River, DE, to St. Augustine, FL (sites listed in Table 1), from 2010–2013. No specific permits were required for the described field studies. The field studies did not involve endangered or protected species. Docks that were used for collections were either private or public marinas. Permissions were sought from the owners or dock masters prior to collecting. All samples were pulled from docks by hand and thus represent animals found close to the water's surface. In many cases, colonies were chosen haphazardly. In some instances, the Type N or S phylotype was targeted for specific investigation, a direction made possible by slight morphological differences observable within multiple mixed *B. neritina* populations (see Fig. 1 for visual representation). A small number of samples were also gathered along the coast of California, at a depth of ∼8 m near Catalina Island by SCUBA (GPS coordinates: latitude 33.346°, longitude −118.331°) and from floating docks in Bodega Bay (GPS coordinates: latitude 38.334°, longitude −123.049°).

**Table 1:** *Bugula neritina* symbiont frequency along North American Atlantic coast.

	Symbiont occurrence				GPS Coordinates	
	Total Type N		Total Type S			
Location	Sym+	Sym−	Sym+	Sym−	Latitude (°)	Longitude (°)
Indian River, DE	0	31	-	-	38.612	−75.072
Ocean Pines, MD	0	22	0	2	38.386	−75.130
Chincoteague, VA	0	8	-	-	37.920	−75.405
Wachapreague, VA	0	9	-	-	37.604	−75.687
Oyster, VA	0	60	10	18	37.289	−75.923
Rudee Inlet, VA	0	19	-	-	36.832	−75.976
Beaufort, NC	-	-	49	1	34.717	−76.666
Radio Island, NC	7	4	35	0	34.714	−76.680
Morehead City, NC	3	0	32	0	34.720	−76.707
Salter Path, NC	9	4	0	1	34.690	−76.887
Murrell's Inlet, SC	-	-	11	0	33.557	−79.031
Isle of Palms, SC	-	-	22	0	32.823	−79.730
Beaufort, SC	13	0	22	0	32.466	−80.666
Hunting Island, SC	12	0	2	0	32.346	−80.469
Tybee Island, GA	-	-	12	2	31.991	−80.852
Jacksonville, FL	-	-	10	0	30.299	−81.413
St. Augustine, FL	-	-	10	0	29.946	−81.307

Symbiont occurrence was determined both for colonies collected haphazardly and in targeted sampling (see Materials and Methods); not all haphazardly selected colonies were assayed for symbiont presence. Dashes indicate host phylotypes not found in a location during sampling.

For samples subjected to DNA analysis, care was taken after collection to ensure that the gathered zooids represent a single colony rather than multiple associated individuals. Distal zooids were cut from several branches in order to avoid ovicells in more proximal reproductive zooids. In several cases, larvae were also collected as they were released by the colonies in subsequent days (variously for both individual colonies and pooled adult samples). When colonies were not immediately subjected to DNA extraction, zooids were cut and stored in RNALater RNA Stabilization Reagent (Qiagen, Valencia, CA) or 70–100% ethanol at −20°C. Similarly, larvae not immediately processed through DNA or chemical extraction were stored in RNALater or 100% methanol for later analysis, or were fixed for fluorescence *in situ* hybridization (FISH; see below). For both adult and larval samples, DNA was extracted using either ZR Fungal/Bacterial DNA MiniPrep or MicroPrep (Zymo Research, Irvine, CA), or DNEasy Blood and Tissue Kit (Qiagen), and following the manufacturer's instructions. Quality and quantity were assessed by spectroscopy (NanoDrop 1000, Thermo Fisher Scientific, Wilmington, DE).

### Molecular characterization of samples

Polymerase chain reaction (PCR) amplification of the mitochondrial COI gene was carried out using the universal invertebrate primers LCO1490 and HCO2198 [34] or the *B. neritina*-specific primer pair BnCOIf and BnCOIr (Table 2). Restriction fragment length polymorphism (RFLP) analysis was performed on amplicons using the restriction endonucleases *Dde*I, which only digests Type S COI products, or *Hha*I, which cuts amplicons from Type N individuals. In order to identify host phylotypes not documented amongst the individual adult colonies collected, PCR was also performed on DNA from the pooled larvae of a large number of colonies (>200) using primers targeting polymorphisms in the Type N and S COI genes (Table 2). The presence of the symbiont “*Ca.* Endobugula sertula” was assessed in DNA by PCR targeting its 16S ribosomal RNA gene (primers EBn16S_254f and EBn16S_643r, Bn240f and Bn1253r [15]), or the bryostatin biosynthetic gene *bryS* [29] (primers BryS_576f and BryS_774r). PCR amplicons and restriction digested products were visualized after agarose gel electrophoresis.

**Table 2:** Primers and probes used in this study.

Name	Sequence	Target	Source
LCO1490	GGTCAACAAATCATAAAGATATTGG	Invertebrate COI	[34]
HCO2198	TAAACTTCAGGGTGACCAAAAAATCA	Invertebrate COI	[34]
BnCOIf	ACAGCTCATGCATTTTTA	*B. neritina* COI	This study
BnCOIr	CATTACGATCGGTTAGTAG	*B. neritina* COI	This study
Bn_COI_N_129f	CACCGGTAGAGATAAAAGTAAT	Type N *B. neritina* COI	This study
Bn_COI_N_615r	CGAATTAAGACAACCTGGTAGT	Type N *B. neritina* COI	This study
Bn_COI_S1_129f	CACTGGTAAAGATAAAAGTAAC	Type S *B. neritina* COI	This study
Bn_COI_S1_615r	AGAATTAAGACAACCAGGCAGC	Type S *B. neritina* COI	This study
Bn240f	TGCTATTTGATGAGCCCGCGTT	“*Ca.* Endobugula sertula” 16S	[15]
Bn1253r	CATCGCTGCTTCGCAACCC	“*Ca.* Endobugula sertula” 16S	[15]
EBn16S_254f	TACTCGTTAACTGTGACGTTACTC	“*Ca.* Endobugula sertula” 16S	[57]
EBn16S_643r	ACGCCACTAAATCCTCAAGGAAC	“*Ca.* Endobugula sertula” 16S	[57]
1055F	ATGGCTGTCGTCAGCT	Eubacterial 16S	[58]
1492R	TACGGYTACCTTGTTACGACTT	Eubacterial 16S	[59]
EBn16S_621f	CCTTAGAGTTCCCAGCCAAAC	“*Ca.* Endobugula sertula” 16S (for ITS sequencing)	This study
ITS-23S-r2	TSTGRDGCCAAGGCATCCA	Eubacterial 23S (for ITS sequencing)	After [60]
BryS_576f	CATTGACAGTCAGTTCTTCATTGA	*bryS*	This study
BryS_774r	CTTTTCCAGATTGAGTTTTTAACCA	*bryS*	This study
Eub338	GCTGCCTCCCGTAGGAGT	Eubacterial 16S	[35]

For selected samples, the COI or 16S PCR amplicon was purified using the GeneJET PCR Purification Kit (Thermo Scientific, Pittsburgh, PA) and sequenced at the Georgia State University Core Facility. An additional PCR was performed for a number of samples using the primers EBn16S_621f and ITS-23S-r2 to amplify a contiguous fragment of the ribosomal RNA gene containing the 3′ end of the 16S rRNA gene (for identity confirmation) and the complete internal transcribed spacer (ITS) region leading to the 5′ end of the 23S rRNA gene. These products were purified with the GeneJET kit and sequenced at Yale University's DNA Analysis Facility (New Haven, CT). Additionally, clone libraries of 16S rRNA gene amplicons generated using the universal primers 1055f and 1492r were constructed for four adult *B. neritina* colonies using the Invitrogen TOPO TA Cloning Kit for Sequencing (Life Technologies, Carlsbad, CA), with 164 clones sequenced at Virginia Commonwealth University's Nucleic Acid Research Facility. Colonies included two Type S from Oyster, VA, determined to be aposymbiotic by PCR analysis, one symbiotic Type S from Oyster, and one symbiotic Type N from Salter Path, NC. Clone identities were determined by BLAST searches, and the proportions of “*Ca.* Endobugula sertula” clones obtained from each sample were analyzed using Fisher's Exact Test.

### Chemical extraction and analysis of *B. neritina* larvae

Crude extracts were obtained from larvae stored in methanol using a six-step process of exhaustive extraction, with the first and last steps using 100% methanol, and the remaining four using an approximately 1∶1 mixture of methanol and dichloromethane as in [18]. Solvents were removed by rotary evaporation and extracts were dissolved in a 6∶3∶1 mixture of methanol:dichlormethane:water according to the number of larvae collected, based upon counts conducted at the time of collection or immediately after extraction. Extracts were analyzed via high-performance liquid chromatography (HPLC, with photodiode array detection, Shimadzu, Columbia, MD) eluted on a C18 analytical column (250×4.6 mm Gemini 5 µm, Phenomenex, Torrance, CA) with a gradient time program using water and acetonitrile as solvents. Chromatogram peaks were examined for maximum absorbance at or near 229 nm, a wavelength previously demonstrated to be diagnostic for bryostatins found along the Atlantic coast of the United States [18].

### Fluorescence *in situ* hybridization (FISH)

Larvae designated for FISH were fixed for 1 h in 4% paraformaldehyde in MOPS-NaCl buffer, then stored in 70% ethanol at −20°C. Hybridization of larvae followed the protocol previously described [13] using a Cy3-labeled universal eubacterial probe (Eub338, [35]) on a subset of larvae, and a Cy3-labeled “*Ca.* Endobugula sertula”-specific probe (Bn1253r) for the rest of the larvae (Table 2). Briefly, to prepare for hybridization, larvae were rinsed in phosphate buffered saline (PBS) and then transferred to hybridization buffer. Probes were added to obtain a final concentration of 5 ng/µl. After incubation at 46°C for 3–4 h, all samples were rinsed in wash buffer and incubated twice in wash buffer for 20 min at 48°C. Larvae were finally washed in PBS with 0.1% Tween-20 and stored at 4°C until observation. Samples were mounted with Vectashield (Vector Labs, Burlingame, CA) and then viewed using an Olympus FluoView FV1000 confocal microscope, with images composed of stacks of 60–80 optical sections 1 µm apart. All images were captured with identical laser intensity and gain settings.

## Results

### Distribution of Types N and S *B. neritina*

**Figure 2:**
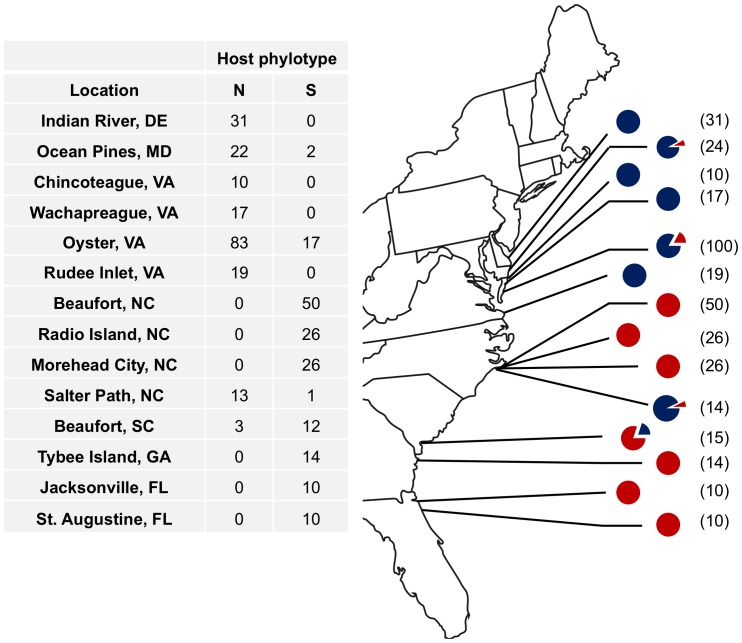
*B. neritina* sibling species collected by haphazard sampling. Proportions at each site indicated by blue (Type N) and red (Type S) in charts.

While *B. neritina* sibling species were largely confined to their initially proposed biogeographic ranges, haphazard colony collection revealed at least four locations in which the atypical species are found among the populations (Fig. 2). To the north of Cape Hatteras, two locations (Ocean Pines, MD, and Oyster, VA) included Type S animals. The Oyster location was extensively sampled (n = 100) and had the largest proportion of Type S animals (17%) among high-latitude locations. These docks were later targeted for Type S colonies, and they were easily found among the Type N animals using observable morphological differences (Fig. 1). Type S COI sequences obtained in this study were identical to the Southern clade of McGovern and Hellberg [26] over the 624 overlapping bases and showed 99.8% identity to the Type S COI sequence of Davidson and Haygood [12], differing by just 1 bp along 483 bases. Type N COI sequences were identical to that of the Northern clade reported in McGovern and Hellberg [26].

**Figure 3:**
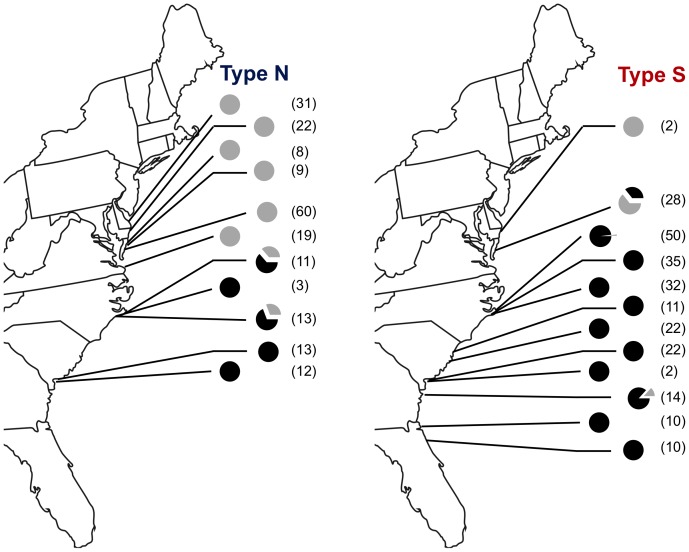
Symbiotic status of sampled colonies. Proportions at each site indicated by black (symbiotic) and gray (aposymbiotic) in charts. Results from both haphazardly collected and targeted samples are included. Number of colonies sampled shown beside each graph.

**Figure 4:**
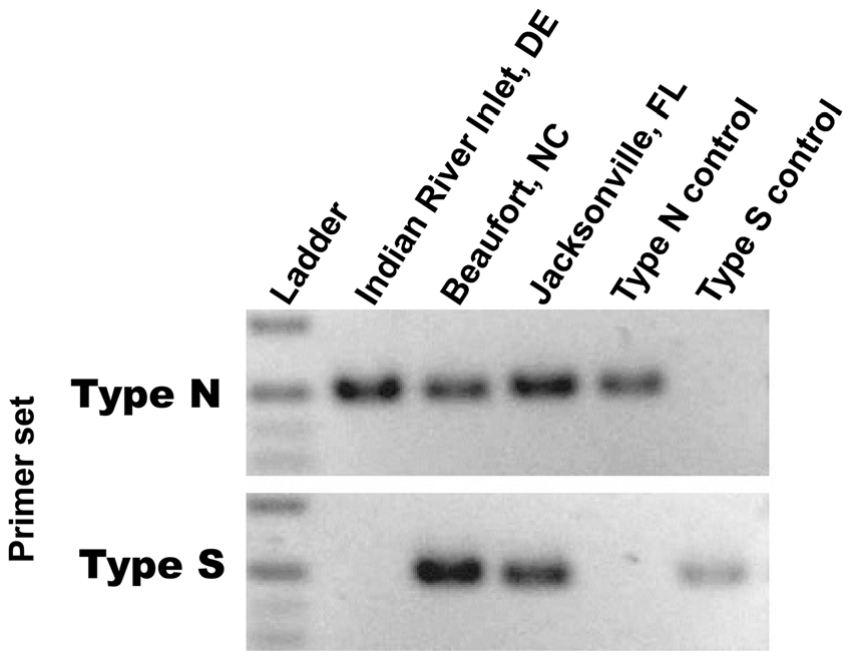
*Bugula neritina* sibling species-specific PCR. Reactions were performed with gDNA (10 ng) extracted from pooled larvae collected from >200 adult colonies as template. Control DNA was extracted from adult *B. neritina* colonies of both phylotypes to serve as 100% Type N or S cytochrome *c* oxidase templates.

As expected, a majority of haphazardly collected *B. neritina* found south of Cape Hatteras were of the Type S sibling species, with only the sites in Beaufort, SC, and Salter Path, NC, including Type N animals. However, collection efforts targeting Type N adult colonies revealed their presence among Type S colonies at a number of other sites: Morehead City, NC; Radio Island, NC; and Hunting Island, SC (Fig. 3). In addition, sibling species-specific PCR using DNA from pooled larvae from a large number of adults (>200) as template revealed the presence of Type N animals as far south as the coast of Florida (Fig. 4).

### Distribution of “*Ca.* Endobugula sertula”

A vast majority (94%) of colonies collected north of Cape Hatteras tested negative for the presence of “*Ca.* Endobugula sertula” (Fig. 3). This was especially true for Type N animals, for which no symbiotic colonies were found. In contrast, our collection revealed 95% of colonies to be symbiotic when located south of Cape Hatteras. Just four aposymbiotic Type S colonies were found south of Cape Hatteras (2%). Type N *B. neritina*, similarly, was largely symbiotic when south of the Cape (85%). This study thus reveals two previously undescribed host-symbiotic status pairings of *B. neritina*: symbiotic Type N and aposymbiotic Type S.

**Figure 5:**
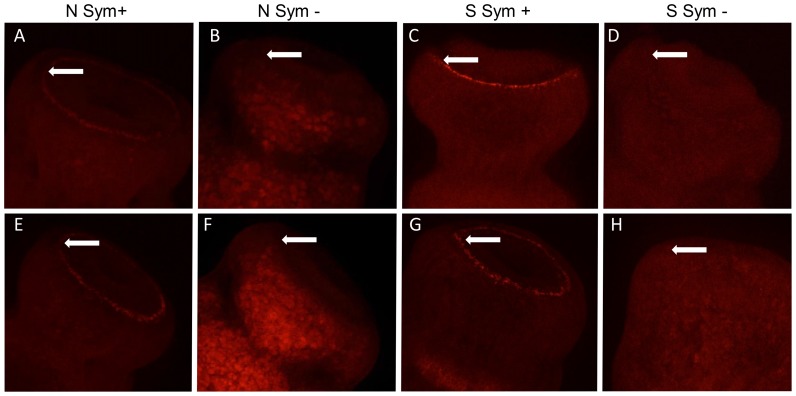
Confirmation of *B. neritina* symbiotic status via fluorescence *in situ* hybridization (FISH). White arrows indicate the circular pallial sinus on the aboral surface of the larva. (A–D) FISH micrographs of larvae using the Eub338 probe. (E–H) FISH micrographs of larvae using the symbiont-specific Bn1253r probe. Diameter of pallial sinus is approximately 150 µm.

The existence of these groups was confirmed by FISH (Fig. 5), as molecular probes specific to “*Ca.* Endobugula sertula” as well as to eubacteria demonstrate the presence of the endosymbiont in the pallial sinus of both Type N and S *B. neritina* larvae which were designated symbiotic by PCR. Similarly, FISH probes failed to reveal the endosymbiont in Type S and Type N animals determined to be aposymbiotic by PCR. Presence of the endosymbiont in larvae collected solely from low-latitude Type N *B. neritina* was additionally demonstrated by PCR targeting the “*Ca.* Endobugula sertula” *bryS* gene, further confirming transmission of symbiont from adult colonies to the next generation (data not shown).

Analysis of 16S rRNA amplicons generated using eubacterial universal primers from adult *B. neritina* colonies (two aposymbiotic Type S, one symbiotic Type S, one symbiotic Type N) revealed the presence of “*Ca.* Endobugula sertula” DNA among clones derived from symbiotic animals (8/85 clones), but none among those constructed using aposymbiotic animals (0/79). The proportion of clones matching the endosymbiont were significantly different for animals identified as symbiotic and aposymbiotic by the PCR assay (*p* = 0.007, two-tailed Fisher's Exact Test).

### Bryostatins associated with Type N “*Ca.* Endobugula sertula”

**Figure 6:**
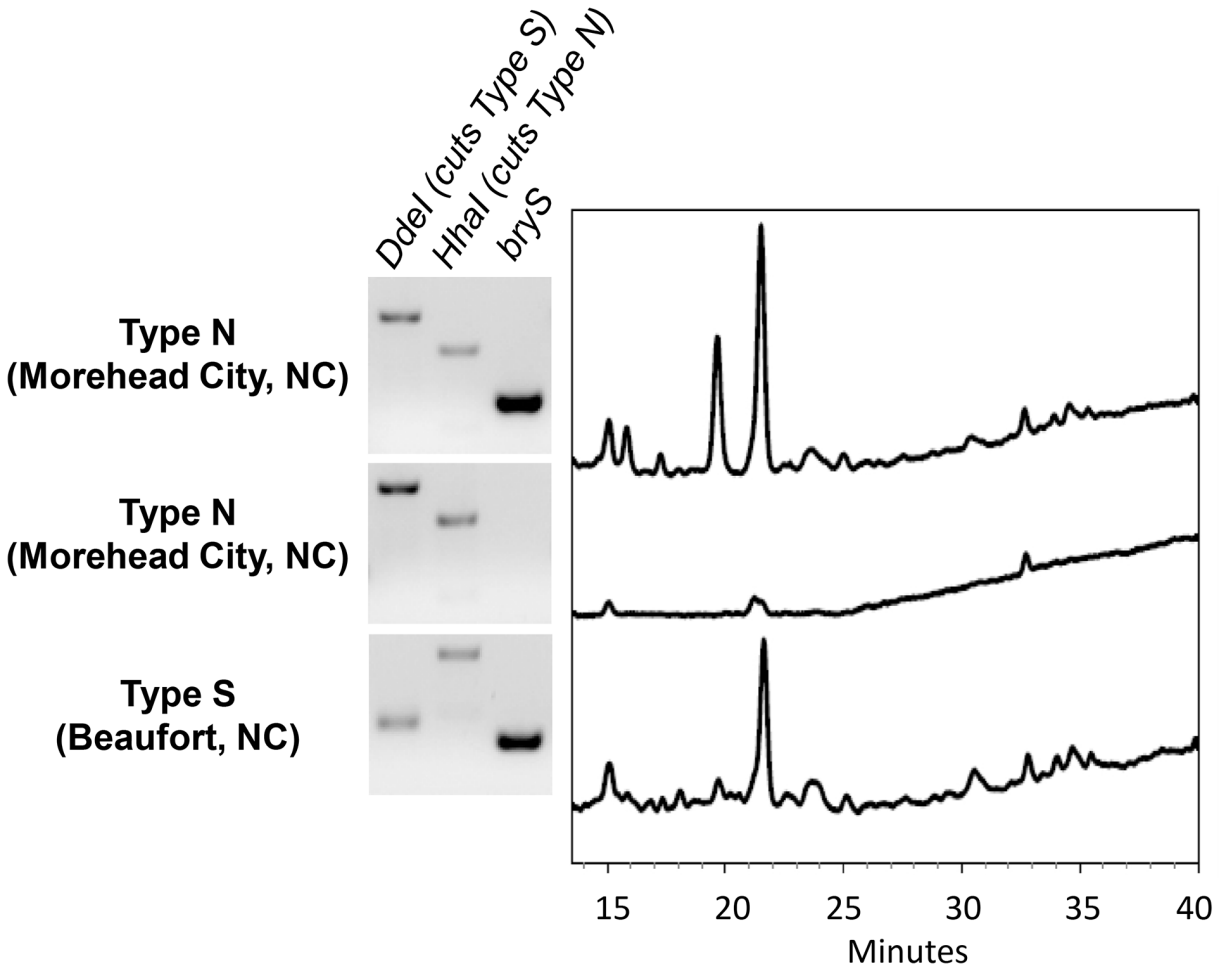
Molecular characterization of *B. neritina* and HPLC analysis of crude larval extract. Agarose gel showing *Dde*I and *Hha*I restriction digestion of *B. neritina* adult (top two samples) or pooled larval (bottom sample) cytochrome *c* oxidase I; amplification of *bryS* to demonstrate presence of symbiont; and absorbance at 229 nm (which is diagnostic of bryostatins) of extract collected from larvae represented by this gDNA.

Analysis of bryostatin composition in Type N symbiotic larval extracts by HPLC at 229 nm illustrates a chemical profile that is very similar to that of pooled larvae from largely Type S populations (Fig. 6). Interestingly, larvae from aposymbiotic Type N *B. neritina* found alongside symbiont-protected Type S animals in North Carolina showed few peaks that appear to be bryostatins when analyzed in this manner. While the co-occurrence of these protected and unprotected colonies is somewhat surprising, the stark chromatographic contrast between symbiotic and aposymbiotic Type N animals highlights the relative similarity of symbiotic Type N larvae to Type S larvae from both Beaufort and Morehead City, NC.

### Sequence analysis of Type N “*Ca.* Endobugula sertula”

Amplicons of the 16S rRNA gene from Type N symbionts collected from Radio Island, NC, Beaufort, SC, and Hunting Island, SC, had sequences identical to those of Type S symbionts from Hunting Island, SC, and Beaufort, SC. These sequences also matched the 16S rRNA gene sequence reported by both Davidson and Haygood [12] and McGovern and Hellberg [26] for Type S “*Ca.* Endobugula sertula”. Sequences obtained using primers starting at position 621 in the 16S rRNA gene and ending in the 5′ region of the 23S rRNA gene also matched previous 16S sequences. An ITS region of ∼850 bp was amplified and demonstrated to be identical between the Type N and Type S symbionts examined. After verification of contiguity from the “*Ca.* Endobugula sertula” 16S sequence to its newly characterized ITS sequence, further sequencing was performed from the 3′ end of the amplicon (starting in the 23S region). All six samples (3 Type N, 3 Type S) were identical across 932 bp of shared sequencing.

Significantly, this identity is in stark contrast to Type D ITS sequences obtained from California colonies. Restriction analysis of host COI amplicons with *Msp*I indicated that all California samples from Catalina Island (n = 7) were of the Type D sibling species, with further digestion of 16S rRNA gene amplicons using *Nhe*I [12] revealing the endosymbiont to also be of Type D. Sequences from two Type D ITS amplicons differed from both the Type N and Type S ITS by 4.0% and 4.3% (Table 3) depending upon the length of quality read. Among *B. neritina* samples taken from Bodega Bay, one proved to be of sufficient quality for DNA characterization. This colony was confirmed to be Type S by COI restriction digestion with *Dde*I and *Msp*I. The ITS sequence amplified from this sample was identical to those of Type S and N “*Ca.* Endobugula sertula” from the Atlantic coast, reducing concerns that the differences between the Type D symbiont ITS and that of Types N and S are a consequence of location. Instead, endosymbiont identity appears to consistently relate to host phylotype.

**Table 3:** Percent identity of marker sequences among *B. neritina* sibling species.

	Host	Symbiont	
% identity between sibling species	COI	Small subunit 16S	ITS
D vs. S	91.8	99.6	95.7
D vs. N	89.6	99.6	95.7
N vs. S	88.5	100	100

Types D and S show the greatest COI identity, but Type D differs from both Types S and N in symbiont markers.

## Discussion

Recent research has revealed that interspecies interactions that are shown to be beneficial in one context may shift to a negative relationship in another where either biotic or abiotic factors vary. For instance, the relationship between cleaning gobies and longfin damselfish is beneficial in high densities of damselfish ectoparasites, but shifts to a more parasitic interaction at low ectoparasite densities [36]. For symbiotic participants that inhabit a wide geographic region, it may be necessary to assess the interaction in different regions to fully understand the dynamic nature of the relationship.

For Western Atlantic *B. neritina* populations, previous sampling had indicated biogeographic separation between two sibling species that varied in their symbiotic status [12], . It was proposed that the aposymbiotic Type N *B. neritina*, which was documented only at higher latitudes, did not possess a defensive symbiont because of lower predation pressures, and that the Type S colonies were symbiotic because they inhabited areas of higher predation pressure. During the course of our study, we discovered that the distribution of the host was much broader than previously thought, while that of the symbiont appears to be restricted by biogeography, instead of host phylotype, as previously thought.

### Ranges of *B. neritina* sibling species

Both Types N and S *B. neritina* can be found across a much wider range of the United States' Atlantic coast than initially observed [26]. This is the second report of these sibling species co-occurring, as well as of their occurrence on both sides of Cape Hatteras [28]. Both symbiotic Type N and aposymbiotic Type S colonies were unexpected findings in this study. The existence of these previously undescribed types may have been a crucial factor enabling the spread of each sibling species beyond the ranges initially understood. Additionally, while Cape Hatteras is not a strict biogeographic boundary for these sibling species, there is still a marked inversion in relative prevalence occurring around this latitude. Physiological factors likely favor Type N *B. neritina* at higher latitudes and Type S at lower, although population structure may be based both on fitness in and initial settlement of a region. Sequencing completed thus far on conspecific colonies from opposite sides of the Cape has not revealed separate lineages within sibling species that are differentially adapted to varying latitudes. Although *B. neritina* larvae have a very short pelagic phase (0.5–2 hr), suggesting a limited dispersal [37], [38], adult colonies are often found on the hulls of ships [39]–[42] and are thought to be dispersed anthropogenically [27], [43]. This transport should result in widespread availability of all haplotypes in many locations, such that the documented population structure is the result of a marked selection pressure.

Details of *B. neritina* reproduction are not well understood, and the possibility of hybridization between these sibling species has not been adequately addressed. If hybrids exist in the environment, one would expect the sibling species' ranges to be obscured, as some individuals may benefit from adaptations originating within the sibling species different from that of the individual's COI gene. For example, apparently Type N animals may persist at lower latitudes due to genes from the Type S lineage, and vice versa. It should also be noted that if “*Ca.* Endobugula sertula” is exclusively vertically transmitted, it is likely that its inheritance would correlate with mitochondrial transmission. Heritable bacterial symbionts are typically inherited maternally [44], [45], though exceptions exist (see [46]). In addition, researchers recently characterized both nuclear and mitochondrial genes from the *B. neritina* species complex worldwide and found no evidence of hybridization, and suggested that these sibling species be regarded as distinct biological species [28]. Thus, it is unlikely that hybridization between Types N and S *B. neritina* is complicating interpretation of the data regarding horizontal transmission of “*Ca.* Endobugula sertula”.

### Ecology of “*Ca.* Endobugula sertula”

The distribution of the endosymbiont “*Ca.* Endobugula sertula” in both Type S and Type N hosts and the similar chemistry associated with these symbionts suggest that their role in defending *B. neritina* larvae applies to both sibling species. Regardless of host identity, symbiosis is more common at lower latitudes, perhaps reflecting the higher predation pressure in regions nearer to the equator [33], [47]. While biogeographic structuring has been noted in marine endosymbionts [48], the latitudinal variation in *B. neritina*-“*Ca.* Endobugula sertula” symbioses may have novel implications for the bryozoan host. For example, symbiosis is more likely to be observed in Type S colonies on both sides of Cape Hatteras, possibly indicating that that they more readily associate with “*Ca.* Endobugula sertula” than do Type N *B. neritina*. It is notable that hybridization may obscure this tendency, as hybrid animals could be falsely identified as strictly one or the other sibling species. However, it is alternatively possible that Type S colonies' stronger association with the endosymbiont is merely a function of greater adaptation to lower latitudes, at which the “*Ca.* Endobugula sertula” is thought to be more beneficial. It is noteworthy that another bryozoan genus, *Watersipora*, also demonstrates both latitudinal variation in genotype [49] and association with putatively defensive symbiotic bacteria [50], but it is unknown if these factors are interrelated as they seem to be for *B. neritina*.

The sympatric association of symbiotic and aposymbiotic colonies at some locations may also be a reflection of the symbiont's impact on host survivorship. The co-occurrence may be briefly observed with the presence of a small number of short-lived colonies, before selection is able to eliminate poorly adapted lineages. However, the occurrence of unexpected associations with “*Ca.* Endobugula sertula,” and the lack thereof, may indicate a previously unexplored cost associated with harboring the symbiont. The value of any facultative symbiosis is necessarily context-dependent [51]. Evaluation of cost within the dynamics of the lifestyle of one or more generations of participants may require observation of a host across its lifetime [6], and may be further complicated by genetic variation within the host, as in the association of the protective symbiont *Hamiltonella defensa* with the black bean aphid [4]. These may be additional factors driving the differing association of Type N and Type S *B. neritina* with their bacterial symbiont.

In addition to defining the range of the symbiosis, the cost-benefit variation of the *B. neritina*-“*Ca.* Endobugula sertula” relationship across space may, in fact, be a force behind the bryozoan's abandonment of association with the bacteria. Mutualism breakdown may be a common result of association across a variety of symbiotic systems [52]. A mode of symbiont loss has yet to be demonstrated in this system in nature, leaving open the possibility of mediation by simple selective pressure, perhaps coupled with imperfect inheritance of the bacteria [53]. Alternatively, environmental factors may limit the survival of the endosymbiont in high-latitude waters, with unprotected aposymbiotic animals found only transiently at low latitudes due to anthropogenic dispersal of adult *B. neritina*. The goal of current experiments is to determine the parameters defining successful maintenance of the association with “*Ca.* Endobugula sertula”.

### Type N “*Ca.* Endobugula sertula”

Analyses using 16S rRNA gene and ITS sequences give no indication that the symbiont associated with Type N *B. neritina* differs from that of Type S colonies. This is surprising if “*Ca.* Endobugula sertula” is subject to strict vertical transmission. Endosymbionts associated with Type S and D *B. neritina* have demonstrated differences of up to 0.5% even in the slowly evolving 16S rRNA gene sequence, with host COI sequences varying by 8.2% [12], [26]. In comparison, Type N and Type S COI sequences differ by 11.5%. One would expect this difference to correlate with an equal or greater likelihood of variation in the symbionts' 16S rRNA gene identity, but none is observed. Indeed, multilocus phylogenetic analysis of the *B. neritina* complex indicates that Type N animals form a sister group to the clade containing Types S and D *B. neritina* [28], further suggesting that cospeciation of host and symbiont would lead to greater divergence between Type S and Type N symbionts.

The slow evolution of the 16S rRNA gene, however, makes in-depth comparison difficult. In fact, while the “*Ca.* Endobugula sertula” sequences obtained by McGovern and Hellberg [26] from Type S *B. neritina* differed from those of Haygood and Davidson's [15] Type S sequences by just 2 bp (0.2%) and Type D sequences by 4–5 bp (0.5%), there were an additional 2 bp that were unique to the later Type S sequences and earlier Type D sequences, while not being shared by the first characterized Type S symbionts. The 16S rRNA gene alone may simply be unreliable for elucidating “*Ca.* Endobugula sertula” phylogeny. In contrast, while 16S rRNA gene sequences may not provide a fast enough clock for contrast, differences might still be seen in ITS sequences, as they have been demonstrated to vary up to 9% among clades even within a single bacterial species [54]. Cytochrome oxidase I variation among *B. neritina* sibling species is well within the expected range for congeneric species of invertebrate phyla, which average 11.3% [55].

Coevolution of host and symbiont would lead to the expectation of associated ITS variation. That these differences have not been observed between Type N and Type S “*Ca.* Endobugula sertula” leaves open the possibility of horizontal transmission of the symbiont between these two sibling species. While potentially not the primary mode of symbiont transfer, such events have been observed in other symbionts demonstrating vertical transmission with a high degree of fidelity [56], and we note that co-analysis of *Bugula* host and symbiont phylogenetic topologies reveals the probability of at least one host-switching event in the lineage's past [11]. The observed similarity between Type N and S symbionts may be the result of a very recent horizontal transmission event, potentially enabled by uptake into the colony's funicular system via the gut. Further investigation of the sibling species' invasion patterns and experimentation with horizontal acquisition of their symbiont may shed more light on the history of this mutualism. In the meantime, our results highlight the flexibility and context-dependence of organismal interactions and the importance of investigating these relationships across scales where abiotic and biotic factors may vary.

### Sequence Availability

New sequence data generated in this study are available in GenBank, accession numbers KM246893-KM246900.
